# Delay in the Diagnosis of Osteoarticular Mucormycosis in Diabetic Ketoacidosis: A Learning Opportunity

**DOI:** 10.7759/cureus.82693

**Published:** 2025-04-21

**Authors:** Louis Boohaker, Sahil M Patel, Erin Townsley

**Affiliations:** 1 Internal Medicine, Baptist Health, Birmingham, USA

**Keywords:** diabetes complications, diabetic ketoacidosis, missed diagnosis, mucormycosis, rhizopus

## Abstract

Mucormycosis is a rare, opportunistic, and life-threatening fungal infection caused by fungi of the order Mucorales, which includes *Rhizopus*,* Mucor*,and *Rhizomucor*. This disease occurs in immunocompromised patients such as those with uncontrolled diabetes mellitus, hematologic malignancies, transplants, trauma, burns, or receiving glucocorticoid therapy. Mucormycosis can present in various ways, including rhino-orbital-cerebral, pulmonary, cutaneous, gastrointestinal, and disseminated disease. Osteoarticular mucormycosis, with the exclusion of bone extension from rhinosinusitis, is an exceedingly rare manifestation. Here, we present a 48-year-old female with uncontrolled type 1 diabetes mellitus who presented with diabetic ketoacidosis. During her hospital course, it was discovered that she had fallen in the woods before presentation, which caused her to develop a left knee wound concerning for osteomyelitis. After symptomatic improvement with antibiotics, she was discharged home, only for us to find out afterwards that her wound biopsy culture grew *Rhizopus*; thus, diagnosing her osteoarticular mucormycosis. She unfortunately was lost to follow-up and was never able to get started on the appropriate treatment. We discuss the clinical manifestations, useful diagnostic tools, and treatment of osteoarticular mucormycosis. We also reflect on how our delay in diagnosis led to our patient being discharged without appropriate treatment in hopes of avoiding similar situations in the future.

## Introduction

Mucormycosis is a rare, opportunistic, and life-threatening fungal infection caused by fungi of the order Mucorales, which includes *Rhizopus*, *Mucor*, and *Rhizomucor* [1]. This disease occurs in immunocompromised patients such as those with diabetes mellitus, hematologic malignancies, transplants, trauma, burns, or receiving glucocorticoid therapy [1]. These fungi are ubiquitous around the globe, typically being found in soil, decaying organic matter, compost, and contaminated foods, with some studies suggesting a seasonal variation with more cases being reported in the spring and summer [2,3]. Transmission occurs primarily through inhalation of fungal spores, but cutaneous invasion via direct inoculation to areas of trauma or ingestion through the gastrointestinal tract may occur [2]. Mucormycosis can present in various ways, including rhino-orbital-cerebral, pulmonary, cutaneous, gastrointestinal, and disseminated disease [1]. Osteoarticular mucormycosis, with the exclusion of bone extension from rhinosinusitis, is an exceedingly rare manifestation, with only 34 cases being reported between 1978 and 2014 [4]. It makes up 15.8% of the total bone and joint non-*Aspergillus* fungal infections [5]. Osteoarticular mucormycosis is difficult to diagnose, with an average time to diagnosis of 73 days [4]. This delay in diagnosis makes it imperative to have early clinical suspicion of osteoarticular mucormycosis and ensure that patients are closely followed so treatment can be initiated. Here, we present the case of a patient with uncontrolled type 1 diabetes mellitus and trauma who was found to have osteoarticular mucormycosis of the left knee one month after discharge.

## Case presentation

A 48-year-old female with type 1 insulin-dependent diabetes mellitus was found unconscious outside. She was very somnolent on admission and was unable to provide much history. On initial presentation, her vitals were significant for an elevated blood pressure at 185/79 mmHg, heart rate of 95 beats per minute, respiratory rate of 18 breaths per minute, and saturation of 100% on room air. Physical examination revealed a chronically ill-appearing, thin female in mild distress with dry mucous membranes. Her left knee was notable for a tender open wound with associated swelling and erythema, but no signs of necrosis or drainage. She had intact left knee passive range of motion, but was unable to assess active range of motion due to her somnolence. Initial laboratory work was significant for white blood cell count of 22.4 × 10^9^/L, potassium of 5.4 mEq/L, serum bicarbonate of 4 mEq/L, blood urea nitrogen of 24 mg/dL, serum creatinine of 1.37 mg/dL, blood glucose of 541 mg/dL, and anion gap of 32 mEq/L (Table 1). Arterial blood gas showed a pH of 7.135 and a partial pressure of carbon dioxide of 14 mmHg (Table 1). Urinalysis was positive for ketonuria and glucosuria (Table 1). Per chart review, her hemoglobin A1c was >18.8% two months before presentation.

**Table 1:** Laboratory results. WBC: white blood count; K: potassium; HCO_3_: serum bicarbonate; BUN: blood urea nitrogen; Cr: creatinine; AG: anion gap; ABG: arterial blood gas; pH: potential of hydrogen; pCO_2_: partial pressure of carbon dioxide; UA: urinalysis

Laboratory test (reference range)	Result
WBC (4.5–11.0 × 10^9^/L)	22.4 × 10^9^/L
K (3.5–5.1 mEq/L)	5.4 mEq/L
HCO_3_ (21–31 mEq/L)	4 mEq/L
BUN (5–23 mg/dL)	24 mg/dL
Cr (0.6–1.2 mg/dL)	1.37 mg/dL
Glucose (70–105 mg/dL)	541 mg/dL
AG (5–15 mEq/L)	32 mEq/L
ABG pH (7.35–7.45)	7.135
ABG pCO_2 _(35–45 mmHg)	14 mmHg
UA ketones (negative)	2+
UA glucose (negative)	3+

**Figure 1:**
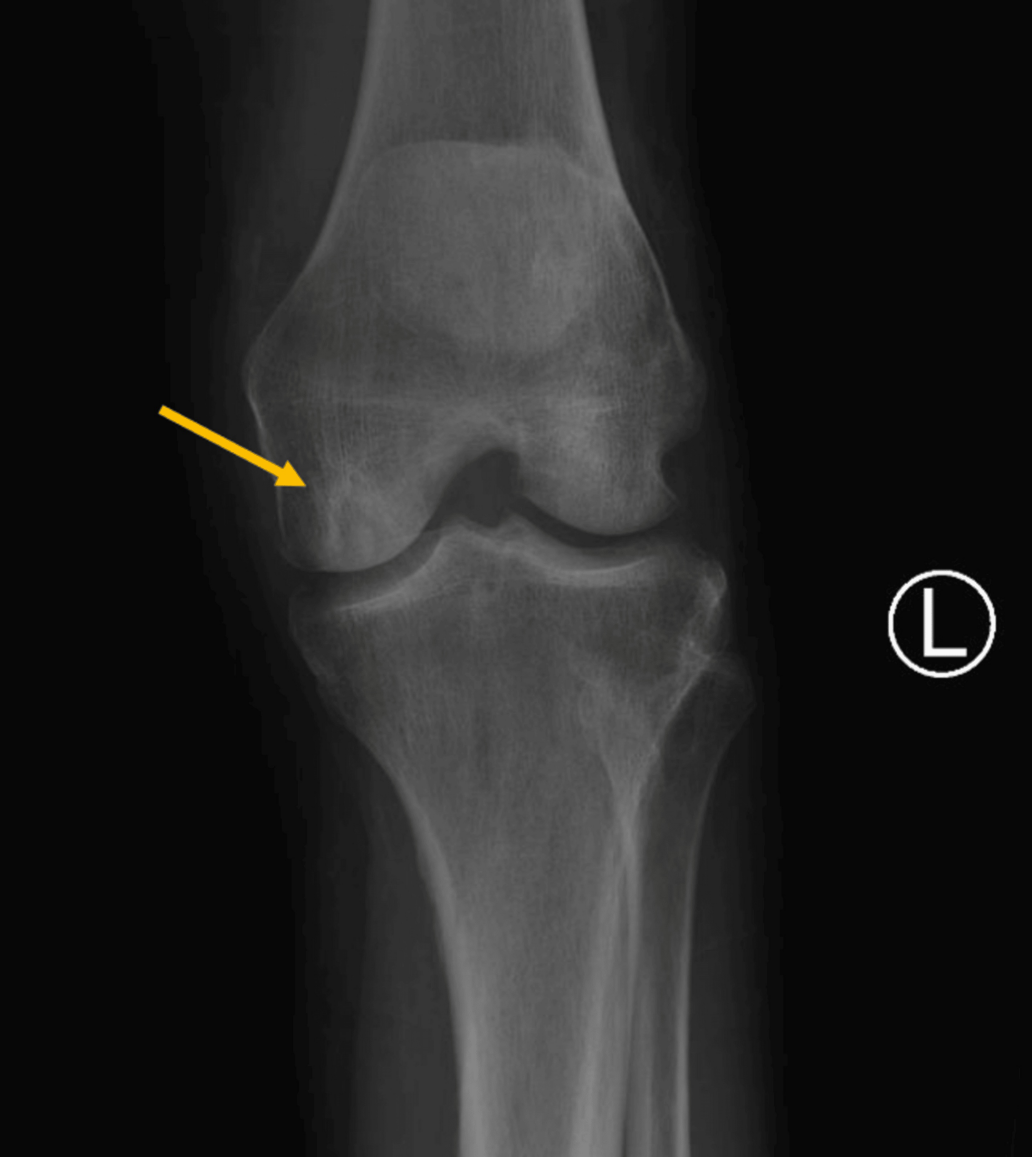
Left knee X-ray depicting a sclerotic lesion in the medial femoral condyle (yellow arrow).

Cephalexin 250 mg four times daily was initiated for presumed left knee non-purulent cellulitis. The diabetic ketoacidosis (DKA) protocol was initiated with the quick resolution of her DKA. As her mentation improved, she was able to provide us with more history. She ran out of her insulin needles and had not been administering any insulin for the past three days, which is why she presented in DKA. She also reported falling in the woods and scraping her left knee sometime in the past week, but was able to provide more information about the specific environment she fell in. However, given the time of the year, it was likely both hot and humid. She described her left knee wound starting as a small lesion but becoming larger and more tender over the last week. A plain film of the left knee was obtained and revealed a sclerotic lesion in the medial femoral condyle (Figure 1). By day three, there was no improvement in her left knee, and she was subsequently broadened to vancomycin, cefepime, and metronidazole to ensure coverage for a polymicrobial infection, including *Pseudomonas aeruginosa*.

**Figure 2:**
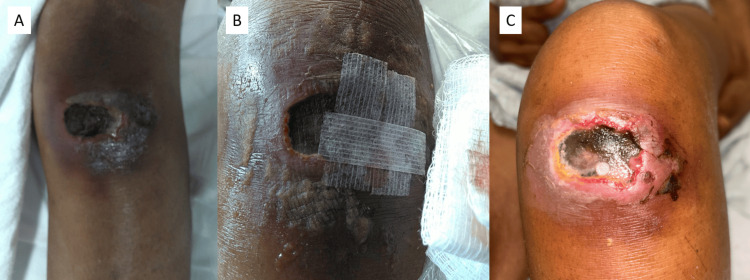
Left knee ulcer following wound debridement showing a 4 cm × 3 cm purple-black ulcer with surrounding erythema and no drainage as seen on hospital day three (A), hospital day four (B), and hospital day six (C).

**Figure 3:**
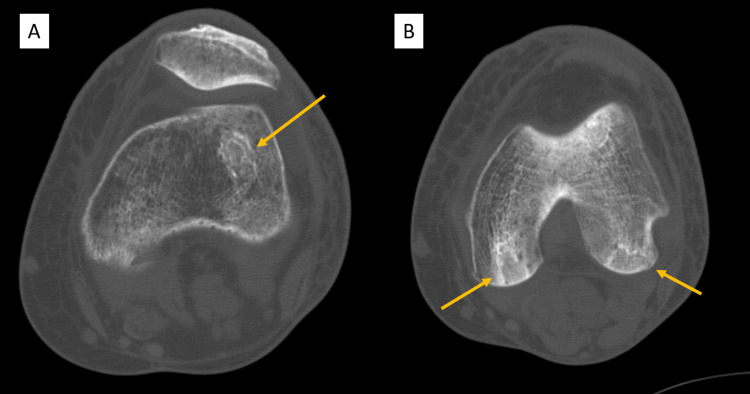
Left knee computed tomography scan without contrast depicting areas suspicious for osteonecrosis in the bilateral femoral condyle (yellow arrows).

Given the possibility of a fungal infection, bedside debridement to mitigate abscess formation and a punch biopsy for fungal culture were performed; however, antifungal treatment was not initiated as a bacterial infection seemed more likely. This revealed a 4 cm × 3 cm purple-black ulcer with surrounding erythema and no drainage (Figure 2A). As the patient had aneurysm clips, she was unable to undergo magnetic resonance imaging (MRI) for investigation of suspected osteonecrosis. As a result, a left knee computed tomography (CT) scan without contrast was obtained and revealed areas suspicious for osteonecrosis in the bilateral femoral condyles (Figure 3). Progression of the wound throughout the hospital course can be seen in Figure 2B (day four) and Figure 2C (day six).

After discussion with orthopedic surgery and radiology, it was agreed that this was most likely cellulitis and that the osteonecrosis seen on the CT scan was likely traumatic avascular necrosis and not related to the infection. She was discharged home with trimethoprim-sulfamethoxazole and given follow-up instructions with our internal medicine residency clinic and orthopedic surgery. A month following discharge, the patient’s fungal culture grew *Rhizopus*, confirming the diagnosis of osteoarticular mucormycosis of the left knee. Unfortunately, she did not show up for her follow-up appointments or fill her prescriptions at discharge. Extensive measures with local authorities were made to get in contact with her, but were unsuccessful. To our knowledge, she had not been admitted to any nearby hospitals, nor was she found at her home address. Given the increased risk of death from hematogenous dissemination, an extensive search online for an obituary was performed, but nothing was found.

## Discussion

Osteoarticular mucormycosis is a rare manifestation of mucormycosis [4]. The pathogenesis is not well understood, but is believed to be due to a functional defect in either macrophages or neutrophils [6]. This is because macrophages inhibit the germination of Mucorales sporangiospores while neutrophils kill proliferating hyphal elements through oxidative burst [7]. In uncontrolled diabetics, such as our patient, there is a known dysfunction of macrophages and neutrophils, which increases the risk of developing mucormycosis [7]. Although uncontrolled diabetes increases the risk of developing osteoarticular mucormycosis, Taj-Aldeen et al. identified that only 18% of osteoarticular mucormycosis cases had a history of diabetes [4]. A history of prior surgery (41%), trauma (21%), or corticosteroid use (21%) was more common [4]. Taj-Aldeen et al. found that 56% of cases involved direct inoculation into the bones and joints [4]. Our patient likely directly inoculated her knee from her fall in the woods, which, coupled with her uncontrolled diabetes, gave *Rhizopus *the perfect setting to flourish. Mucormycosis is characterized by its angioinvasion and localized destruction of tissue, which allows it to disseminate to other locations in the body [3,7]. As a result, it is crucial to identify and start treatment as early as possible to decrease the risk of dissemination.

The clinical features of mucormycosis can vary based on the location of infection [7]. Osteoarticular mucormycosis typically presents with signs of local inflammation, which can include pain and tenderness, fever, swelling, cellulitis/ulcer/abscess, and restricted movement [7]. Osteoarticular mucormycosis, when compared to the more common rhino-orbital-cerebral mucormycosis, tends to remain localized for a longer duration before disseminating [7]. In our case, the patient had pain and tenderness with associated swelling and cellulitis but did not present with fever or restricted movement.

Initial investigation should evaluate for osteomyelitis with X-ray, non-contrast CT, or non-contrast MRI, but this does not help rule out fungal etiology [8]. The gold standard for diagnosis is to obtain a biopsy for microscopy, cell culture studies with Mucorales polymerase chain reaction, and histopathology [9]. The European Confederation of Medical Mycology guideline recommendations strongly recommend antifungal therapy with amphotericin B and surgical debridement, aimed at removing necrotic tissue and thrombosis to aid in antifungal penetration [10,11]. Alternatives to amphotericin B include posaconazole and isavuconazole, which show a higher activity against Mucorales than other triazole antifungals [11]. There are no recommendations in terms of duration of treatment, but studies have shown a wide range in treatment durations from one week to almost three years [10]. Contrast-enhanced MRI and conventional CT scans can be used for monitoring response to therapy [9].

## Conclusions

Given that the diagnosis of osteoarticular mucormycosis takes an average of 73 days, it is crucial to obtain up-to-date contact information and emphasize the importance of follow-up. As seen with our patient, her loss to follow-up ultimately led her to not getting the appropriate treatment for her osteoarticular mucormycosis. Our case highlights the importance of considering osteoarticular mucormycosis in immunocompromised patients presenting with signs or symptoms of osteomyelitis. We recommend including fungal infections on the differential diagnosis for immunocompromised patients. This includes obtaining a biopsy for fungal culture, early on, while taking into consideration the extended time it may take to result. Close follow-up and up-to-date contact information are imperative to avoid a similar circumstance in the future. In addition, emphasizing the dangers of an untreated mucormycosis infection to the patient can help encourage them to follow up.
